# Machine Learning as a Multifaceted Tool in Glomerular Diseases: Beyond Risk Prediction

**DOI:** 10.1159/gdz/acyag004

**Published:** 2026-10-01

**Authors:** Maria J. Vargas-Brochero, Reid P. Schlesinger, Wisit Cheungpasitporn, Charat Thongprayoon, Jing Miao

**Affiliations:** a Division of Nephrology and Hypertension, Mayo Clinic, Rochester, MN, USA; b Department of Medicine, Mayo Clinic, Rochester, MN, USA

**Keywords:** Machine learning, Glomerular disease, Digital pathology, Precision nephrology, Risk stratification

## Abstract

**Background:**

Glomerular diseases are a heterogeneous group of disorders with highly variable clinical outcomes, ranging from spontaneous remission to progressive kidney failure. Traditional prognostic tools, including proteinuria, serum creatinine, and semiquantitative histopathologic scoring, capture only part of this complexity. In particular, they often reduce longitudinal patient data to static baseline measurements, discarding valuable information about disease trajectories, rate of change, and temporal variability. Machine learning (ML) has emerged as a promising analytical framework capable of addressing these limitations by processing time-series data, modeling nonlinear relationships, integrating multimodal data, and generating individualized risk estimates.

**Summary:**

This review introduces fundamental ML paradigms relevant to nephrology, including supervised learning, unsupervised learning, reinforcement learning, and deep learning. A practical ML pipeline for clinical application is proposed, and key data sources that power modern models are introduced. Current clinical applications of ML in glomerular disease are highlighted, including digital pathology for automated histologic assessment, data-driven phenotyping to identify disease subtypes, improved diagnostic differentiation, and enhanced prediction of prognosis and treatment response. Lessons learned from clinical deployment failures, key challenges, ethical considerations, and future directions are also discussed.

**Key Messages:**

ML enables deeper phenotyping and more accurate risk stratification by capturing complex nonlinear patterns in multimodal data that traditional methods cannot. Current applications span digital pathology, diagnostic support, prognostic modeling, and treatment-response prediction, with some tools now incorporated in clinical guidelines. Successful clinical translation requires diverse training data, rigorous external validation, interpretable model architectures, and ongoing collaboration between data scientists, clinicians, and regulators. ML augments, not replaces, clinical expertise – maximizing value from each patient encounter and biopsy specimen to improve individualized care and outcomes.

## Introduction

Glomerular diseases represent a heterogeneous group of disorders marked by highly variable clinical outcomes, ranging from spontaneous remission to irreversible progression toward kidney failure [1]. Traditional prognostic tools, including baseline proteinuria, serum creatinine, and semiquantitative histopathologic scoring, capture only part of this complexity [2]. They typically reduce longitudinal patient data to static baseline measurements, discarding valuable information about disease trajectories, rate of change, and temporal variability. These conventional parameters often fail to account for nonlinear relationships or subtle interactions among clinical, molecular, and histologic factors.

Over the past decade, machine learning (ML), a subset of artificial intelligence, has emerged as a powerful analytical framework capable of addressing these limitations in several key ways [3]. First, to capture dynamic temporal patterns, ML architectures such as recurrent neural networks, long short-term memory networks, and transformer-based models can process time-series data such as longitudinal eGFR trajectories and proteinuria trends to identify trajectory-based risk signatures that traditional static models (e.g., Cox regression using only baseline values) fail to detect. Second, to accommodate nonlinear relationships, tree-based methods (e.g., random forest) and deep learning approaches can model complex, nonadditive interactions without requiring prespecification. Third, to integrate multimodal data, ML frameworks combine clinical, histopathologic, and molecular information into unified risk predictions. Within the specific context of glomerular disease, ML is increasingly being deployed to define outcome risk, uncover latent disease subtypes, analyze high-dimensional imaging and molecular datasets at scale, and support clinical decision-making [4].

Despite rapid methodological advances, a gap remains between predictive performance and clinical utility. Many ML models achieve high discrimination yet offer limited decision support, underscoring the need for a framework that links prediction to action [5]. This review proposes a practical ML pipeline to advance clinical use, introduces the fundamental ML paradigms, and highlights current applications of ML in glomerular diseases through several examples. Lessons learned from clinical deployment failures, challenges and ethical considerations, and future directions are also examined. Our objective was to clarify how ML can augment clinical reasoning and accelerate the development of individualized strategies aimed at improving patient outcomes.

## Proposed ML Pipeline in Glomerular Diseases

To translate ML from promise to practice, a structured framework is needed. Figure 1 presents a proposed ML pipeline for clinical application in glomerular diseases. The pipeline begins by defining the clinical goal along with (1) defining target population and sample inclusion/exclusion criteria to ensure the model addresses a clinically relevant question and minimizes selection bias; (2) defining the primary outcome with clear, reliable ascertainment methods; and (3) selecting the predictor pool based on clinical plausibility, prior evidence, and availability in real-world practice, rather than relying solely on data availability. Only after these steps should data acquisition begin integrating multimodal sources such as electronic health records (EHRs), digital pathology, multi-omics data, and wearable or remote monitoring (Table 1).

**Fig. 1.**
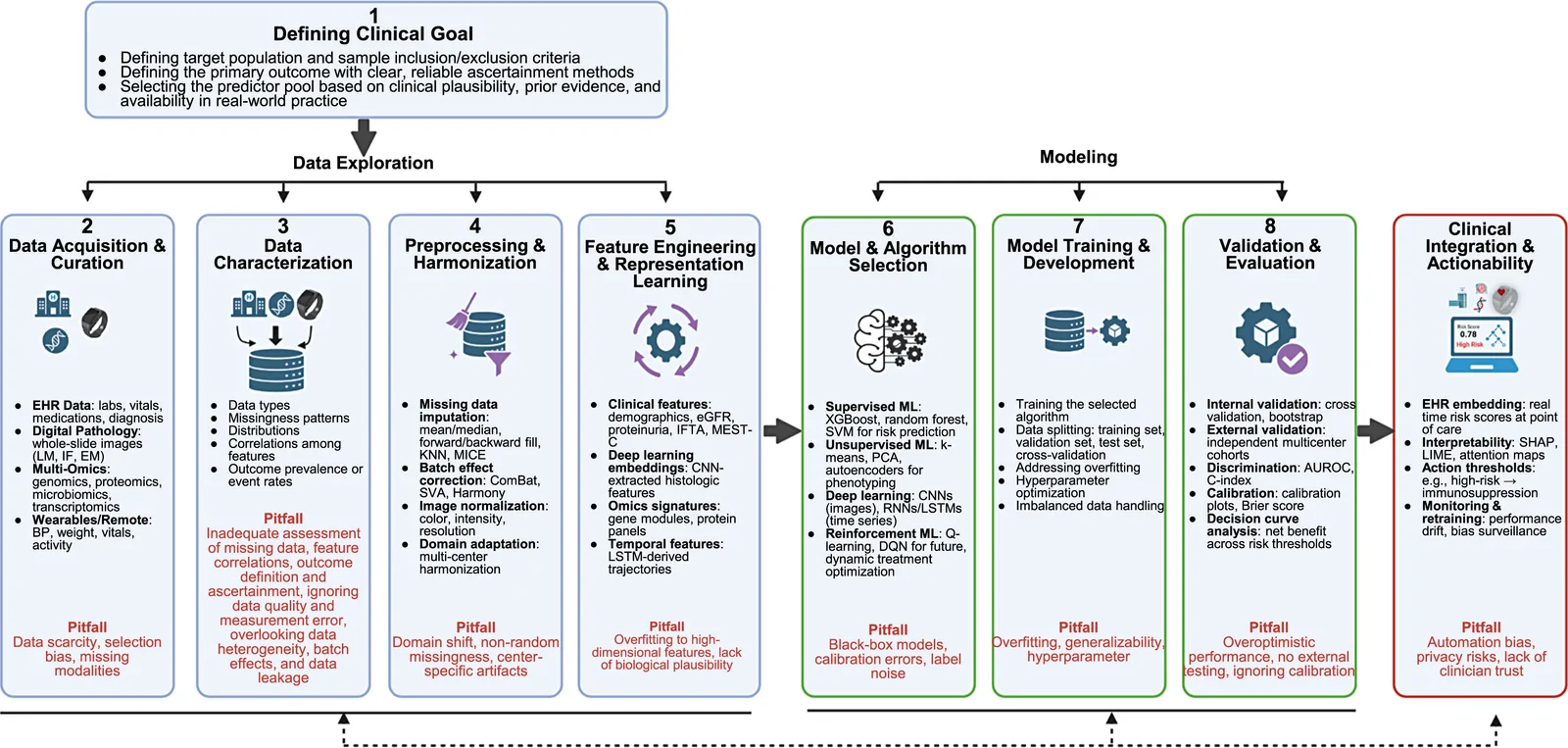
Proposed ML pipeline in glomerular diseases. The pipeline begins with defining the clinical goal, followed by data exploration (data acquisition, characterization, preprocessing, and feature engineering), model development (model/algorithm selection, training, and validation), and integration of validated models into EHR workflows with interpretability, actionability thresholds, and ongoing monitoring for drift or bias. The ML approaches listed (e.g., XGBoost, SVM, PCA, CNN, LSTM, DQN) are illustrative examples only and do not represent an exhaustive or prescriptive list. The appropriate model choice depends on the data characteristics and clinical task. Dashed arrows indicate iterative feedback loops. For example, validation failures may prompt model revision, while deployment-related performance degradation may require data reacquisition or re-preprocessing (created in BioRender. Miao, J. (2026) https://BioRender.com/9v7nd63). ML, machine learning; EHR, electronic health record; SVA, surrogate variable analysis; LM, light microscopy; IF, immunofluorescence; EM, electron microscopy; BP, blood pressure; MICE, multivariate imputation by chained equations; KNN, k-nearest neighbor; eGFR, estimated glomerular filtration rate; IFTA, interstitial fibrosis/tubular atrophy; MEST-C, mesangial hypercellularity, endocapillary hypercellularity, segmental glomerulosclerosis, tubular atrophy/interstitial fibrosis, crescents; CNN, convolutional neural network; LSTM, long short-term memory; XGBoost, eXtreme gradient boosting; SVM, support vector machine; PCA, principal component analysis; RNN, recurrent neural network; DQN, deep Q-network; AUROC, area under the receiver operating characteristic curve; SHAP, SHapley Additive exPlanations; LIME, local interpretable model-agnostic explanations.

**Table 1.** Data sources for machine learning in glomerular disease

Data source	Key content	Advantages for ML	Key challenges	Examples in glomerular disease
Clinical data	EHRs: laboratories (e.g., serum creatinine, proteinuria, albumin, eGFR), vital signs, comorbidities, medications	Provides longitudinal, real-world patient trajectories; readily available; forms the backbone of most prediction models (e.g., eGFR decline)	Non-random missingness; measurement variability; coding noise from inconsistent diagnostic codes	Predicting eGFR slope, risk of disease progression, or response to immunosuppressive therapy
Biopsy-derived information	Digital whole-slide images of kidney tissue; immunofluorescence; immunohistochemistry; EM; traditional histopathological scores (e.g., MEST-C, IFTA)	Offers high spatial resolution of structural and inflammatory injury; computational analysis can automate segmentation and quantify subtle morphologic features invisible to the eye	Requires specialized infrastructure for digital pathology; traditional scoring is coarse; high-dimensional image data requires advanced models (e.g., CNNs) for feature extraction	Automatic detection and classification of glomerular lesions; quantification of fibrosis and sclerosis; discovery of novel histo-phenotypic signatures
Proteomics, genomics, and multi-omic data	Genomic data (e.g., *APOL1* risk variants), transcriptomics, proteomic, metabolomic profiles from urine or blood	Provides mechanistic insight into disease pathways; captures molecular heterogeneity; identifies dynamic biomarkers reflecting real-time disease activity	High dimensionality relative to sample size; batch effects and platform harmonization issues; requires complex normalization and external validation	Integrating genetic risk scores with clinical data; identifying serum or urine protein panels that predict disease activity or treatment response
Wearables and patient-generated data	Continuous data from sensors: blood pressure variability, physical activity, sleep patterns, weight trends, bioimpedance	Captures physiologic fluctuations between clinic visits; enables dynamic, high-frequency monitoring; can potentially detect early signs of disease flare	Data are often noisy and unstructured; requires patient adherence; integration with EHRs is complex; clinical utility in glomerular disease remains speculative	Early detection of volume overload (e.g., weight trends); tracking blood pressure patterns in hypertensive nephropathy; remote monitoring for post-transplant or clinical trial patients
Multicenter cohorts and registries	Aggregated clinical, laboratory, and biopsy data from multiple institutions (e.g., CureGN, Neptune)	Increases sample size and statistical power; enhances diversity and generalizability of models; enables robust training and validation of complex algorithms	Heterogeneity in practice and assays across sites; differences in data collection standards; requires sophisticated harmonization and domain adaptation techniques	Developing externally validated prediction models for rare disease progression; training models robust to different patient populations and clinical settings

eGFR, estimated glomerular filtration rate; MEST-C, mesangial hypercellularity (M), endocapillary hypercellularity (E), segmental glomerulosclerosis (S), tubular atrophy/interstitial fibrosis (T), crescents (C); IFTA, interstitial fibrosis/tubular atrophy; APOL1, apolipoprotein L1.

Following data acquisition, data exploration and characterization is critical. Before any model or algorithm is selected, the data must be thoroughly characterized – including data types, missingness patterns, distributions, correlations among features, and outcome prevalence or event rates. Only after this exploratory phase should appropriate ML approaches be considered as the choice of model depends critically on the data structure (e.g., tabular vs. imaging vs. longitudinal), sample size and event counts, missing data mechanisms, and the clinical prediction task (classification, regression, or time to event).

The subsequent sequential stages are as follows. Data preprocessing and harmonization handles missing data, batch effects, image normalization, data integration, and outlier detection to ensure data quality, consistency, and comparability across samples and institutions. Crucially, preprocessing choices should be documented transparently and, where possible, guided by clinical input to ensure that decisions (e.g., how to handle missing data, which outliers to exclude) are clinically sensible and do not introduce bias. The TRIPOD + AI guidelines provide a framework for reporting preprocessing steps to ensure reproducibility and facilitate critical appraisal [6]. Feature engineering and representation learning converts raw data into predictive features that optimally represent the underlying biology and clinical context while remaining compatible with the chosen modeling approach, with key considerations including selecting and transforming clinical variables based on known prognostic associations; deriving standardized histologic scores and computationally extracted features; capturing longitudinal trajectories (e.g., eGFR slope, proteinuria trends); extracting deep learning-derived embeddings from imaging or other unstructured data; applying feature selection and dimensionality reduction to mitigate overfitting; incorporating clinically plausible interaction terms; and ensuring feature definitions are externally validated and standardized across centers. Clinicians should be actively involved in this step to ensure features reflect meaningful pathophysiology, avoid biologically implausible combinations, and align with clinical reasoning. Model and algorithm selection, whether supervised, unsupervised, reinforcement, or deep learning (Fig. 2), is not a one-size-fits-all decision but should be driven by the characteristics of the data and the nature of the clinical prediction task. Comparing multiple candidate models suited to the data characteristics and selecting the best-performing approach through rigorous cross-validation is recommended, while considering the trade-off between predictive performance and interpretability. Model development involves training the selected algorithm on the prepared dataset, with careful attention to avoiding overfitting, optimizing hyperparameters, and ensuring generalizability. Validation and evaluation require internal validation, external validation on independent cohorts, and assessment of discrimination, calibration, and decision curve analysis (DCA) to determine net clinical benefit.

**Fig. 2.**
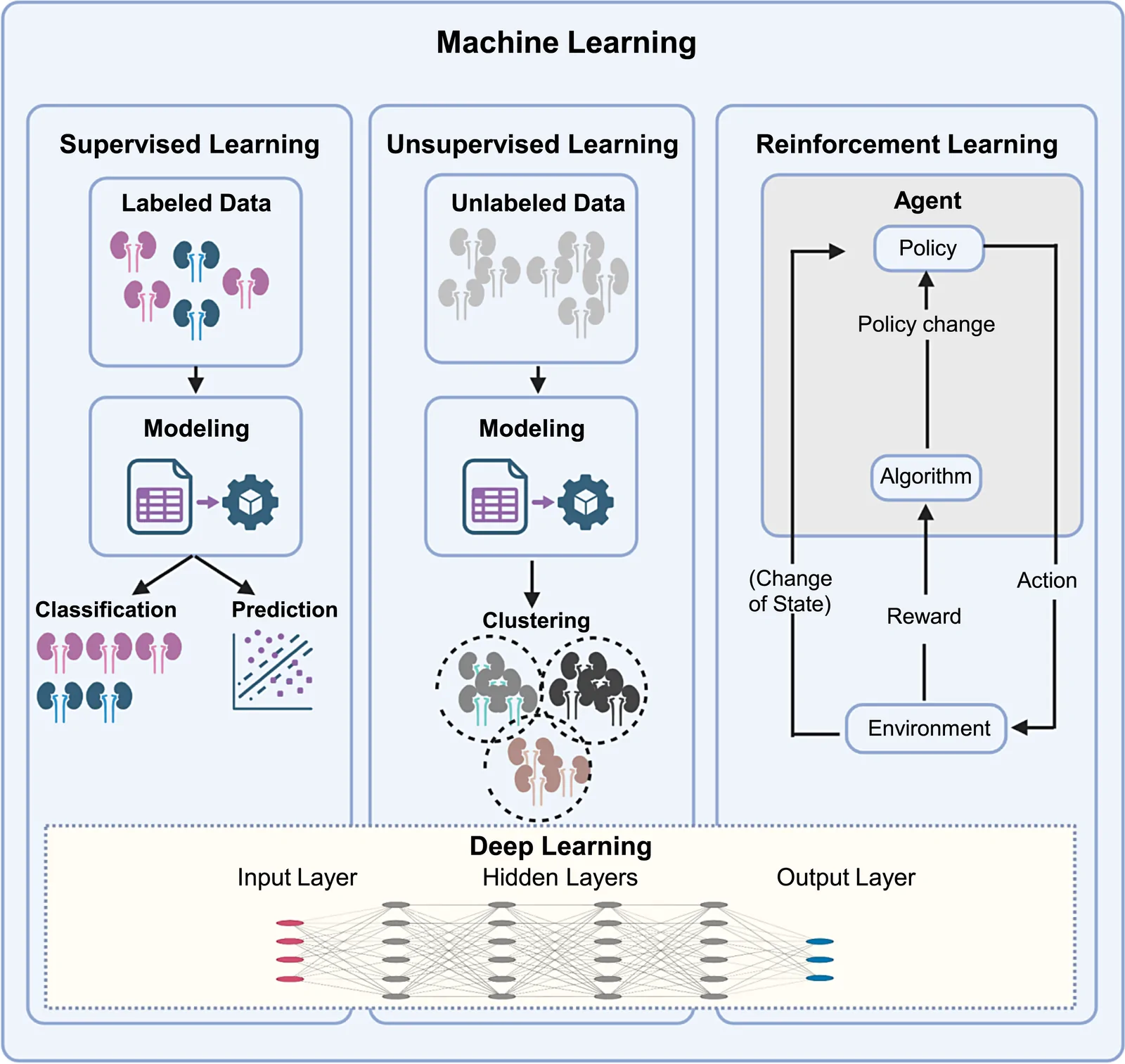
ML paradigms. ML encompasses three primary paradigms, including supervised learning, unsupervised learning, and reinforcement learning, each defined by how the algorithm learns from data. In supervised learning, a model is trained on labeled data to perform tasks such as classification and prediction. In unsupervised learning, a model identifies intrinsic structure, such as clusters, within unlabeled data. In reinforcement learning, an agent interacts with an environment, selecting actions and receiving reward-based feedback to iteratively refine a decision-making policy. Deep learning, built on multilayer neural networks composed of an input layer, hidden layers, and an output layer, is not a separate paradigm but rather a class of methods applicable within each of the three paradigms (created in BioRender. Miao, J. (2026) https://BioRender.com/i2ypkvq).

The final step is clinical integration and actionability, which involves embedding validated models into EHR workflows, ensuring interpretability, setting actionability thresholds for clinical decision-making, and continuously monitoring for performance drift or bias. Importantly, clinical input is critical not only at the outset but throughout the pipeline – from ensuring preprocessing choices are clinically sensible, to validating that model outputs align with clinical reasoning, to designing user interfaces that support rather than disrupt workflow.

## Fundamental Paradigms of ML

ML refers to computational methods that learn patterns from data [7]. The degree of ML autonomy varies across different ML approaches. At a minimum, all ML models learn parameters – such as regression coefficients – from data rather than relying on hand-crafted rules. More flexible methods, including deep learning and tree-based algorithms, also learn feature representations or decision boundaries without requiring humans to prespecify nonlinear interactions or complex transformations [8]. This flexibility makes ML well-suited to glomerular diseases, which exhibit several features that challenge traditional statistical approaches: nonlinear interactions between clinical and histologic variables, time-varying disease dynamics, and high-dimensional multimodal data. ML is broadly divided into three primary paradigms – supervised learning, unsupervised learning, and reinforcement learning – each defined by how the algorithm learns from data (Table 2).

**Table 2.** Comparison of machine learning paradigms

​	Supervised learning	Unsupervised learning	Reinforcement learning	Deep learning
Data type	Labeled data (both input and output)	Unlabeled data (input only, no output labels)	Generates its own data by interacting with an environment, receiving reward or penalty signals	Can handle labeled, unlabeled, or raw data; often thrives on very large-scale datasets
Application	Predict outcomes for new data: classification (categorical), regression (continuous), and time-to-event (survival) accounting for censoring^a^	Explore data to discover intrinsic structures (e.g., clusters, latent representations) without guidance	Learn an optimal policy dictating which actions an agent should take in a given situation to maximize cumulative reward	Automatically learn complex, multi-level features directly from raw data for tasks like classification, detection, generation, or representation learning
Key algorithms	Linear regression^b^ Logistic regression k-nearest neighbors (k-NN) Decision tree^c^ Random forest XGBoost LightGBM SVM	k-means clustering Hierarchical clustering PCA	Q-learning DQN REINFORCE PPO	CNN RNN LSTM Autoencoders Transformers

RNN, recurrent neural network.

^a^ All survival ML methods must properly handle censoring (patients without events by study end or lost to follow-up) and variable follow-up times – pervasive challenges in observational cohorts often inadequately addressed.

^b^ Linear and logistic regression are included as ML methods because they learn coefficients from data, not fixed rules. However, they require analysts to prespecify functional forms (e.g., linearity, additivity) and feature transformations, lacking the autonomous feature learning of deep learning or tree-based methods.

^c^ Tree-based methods are a family of ML algorithms that use decision trees as the fundamental building block for prediction. They work by recursively splitting data into subsets based on feature values, forming a tree-like structure of decisions. Key algorithms include single decision trees (e.g., CART), random forest, gradient boosting (XGBoost, LightGBM, CatBoost), and extra trees. Their advantages include interpretability (visualizable trees), no linearity assumptions, automatic interaction detection, robustness to outliers, and seamless handling of mixed data types.

### Supervised Learning

Supervised learning is the most widely used ML paradigm. In this approach, models are trained on labeled datasets, where input features are paired with known outcomes, allowing the model to learn patterns that can be applied to new, unseen data [7]. Supervised learning typically handles three types of tasks: classification (predicting categories), regression (predicting continuous values), and time-to-event prediction (predicting when an event will occur while accounting for censoring). Time-to-event prediction is especially relevant to glomerular diseases, which are often studied in observational cohorts with variable follow-up times. Common outcomes in this context include time to end-stage kidney disease (ESKD), time to doubling of creatinine, time to renal flare, and time to remission.

Self-supervised learning is a training method where the model creates its own “answer key” from unlabeled data [7, 8]. The model learns by solving a pretext task it generates from the data itself, such as predicting the next sentence in a paragraph or filling in a missing patch of an image. An example of this approach is self-distillation with no labels (DINO).

### Unsupervised Learning

Unsupervised learning analyzes unlabeled data to find hidden structure without using predefined outcome labels [7]. Its common applications include clustering, which groups similar observations together, and dimensionality reduction, which compresses high-dimensional data into a smaller set of informative features for visualization or further analysis.

### Reinforcement Learning

Reinforcement learning is the third major category of ML and differs fundamentally from supervised and unsupervised approaches [7]. Instead of learning from a fixed dataset, an agent learns by interacting with an environment, taking actions, and receiving reward-based feedback to develop a strategy that maximizes cumulative reward over time. In glomerular disease, applications are currently rare and mostly exploratory. However, because reinforcement learning is designed for sequential decision-making, it may 1 day help optimize treatment strategies or assist with procedures such as robotic biopsy guidance.

### Deep Learning

Deep learning is not a separate learning paradigm but rather a family of modeling architectures based on multilayer artificial neural networks (ANNs). These architectures can be trained using supervised, self-supervised, unsupervised, or reinforcement learning objectives to learn complex, hierarchical representations from data [9]. A deep learning network consists of an input layer that receives raw, high-dimensional data, multiple hidden layers that extract increasingly abstract features, and an output layer that produces a prediction or classification. This capability is especially valuable in information-rich settings relevant to glomerular disease. Deep learning often outperforms conventional approaches when training datasets are large and the data contain complex spatial, temporal, or multimodal structure.

## Current Applications of ML in Glomerular Disease

ML is being used more often in kidney research, including glomerular diseases (online suppl. Fig. 1; for all online suppl. material, see https://doi.org/10.1159/gdz/acyag004). However, ML is not always needed. Standard statistics, like regression, work well when relationships are simple, there are few variables, and interactions are known ahead of time. ML becomes useful in three situations: (1) when relationships are complex or nonlinear, such as combined effects of proteinuria, tissue features, and blood markers; (2) when data are high dimensional relative to sample size, like digital images or genetic data; and (3) when the goal is to find hidden patterns without pre-set labels, such as identifying new disease groups.

ML is less helpful when sample sizes are very small relative to the complexity of the model – a concern that depends not on the type of learning and the number of candidate predictors. As a general principle, models with many parameters require larger event counts to avoid overfitting. For example, the classical recommendation of ≥10 events per variable for logistic and Cox regression has been refined by more recent frameworks that also account for anticipated model performance and the event fraction [10]. Tree-based ensemble methods and deep learning architectures generally require larger effective sample sizes to achieve stable performance [11], while unsupervised methods require sufficient samples relative to the feature space to identify robust clusters [12]. Techniques such as transfer learning, data augmentation, and penalized regression can partially mitigate small-sample limitations but do not eliminate them [13].

The decision to use ML should be driven by the research question and data, rather than by the appeal of the technology itself. The following sections highlight selected examples from the literature (online suppl. Tables 1–5). Because this is a narrative review, we prioritized methodologically representative studies that illustrate the range of current applications. A concise description of the search strategy and study selection process is provided in online supplementary Material 1.

### Digital Pathology and Image-Based Assessment

Kidney biopsy remains essential for diagnosing glomerular disease, yet expert nephropathologists are scarce worldwide – particularly in resource-limited settings. ML, especially deep learning, offers a way to support and extend nephropathologists’ capabilities by automating repetitive quantitative tasks. It is important to emphasize that ML cannot substitute for the integrated clinical-pathological reasoning, communication with treating clinicians, or interpretation of atypical and rare cases that define expert nephropathology practice.

#### Automated Detection and Classification

An early study used a convolutional neural network (CNN) on biopsy images from 171 patients to measure fibrosis and predict kidney outcomes [14]. The CNN outperformed pathologist estimates across several tasks. For predicting chronic kidney disease (CKD) stage, the CNN had a Cohen’s kappa of 0.519 vs. 0.051 for pathologists. For predicting high creatinine, the CNN achieved an area under the receiver operating characteristic curve (AUROC) of 0.912 (vs. 0.840) and an F1 score of 0.953 (vs. 0.918). For nephrotic-range proteinuria, the CNN achieved an AUROC of 0.867 (vs. 0.702) and an F1 of 0.720 (vs. 0.446). For 1-, 3-, and 5-year kidney survival (defined as the need for dialysis, renal transplant, or death), the CNN had AUROCs of 0.878, 0.875, and 0.904, respectively, all higher than the pathologist-based model. Although limited by a small sample, a fibrosis-only focus, and lack of external validation, this proof-of-concept study provided early evidence that deep learning has the potential to perform as well as, or better than, pathologist-based prognostic assessment.

Later work expanded on this. One group developed a pipeline to identify glomerular structures from PAS-stained images of patients with diabetic nephropathy (DN) [15]. The method performed well on component tasks: glomerular boundary detection (balanced accuracy 0.93), nuclei detection (sensitivity 0.94, specificity 0.93), and luminal component detection (sensitivity 0.98, specificity 0.99). For disease classification, the approach showed moderate agreement with expert pathologists (κ = 0.55), similar to agreement among pathologists themselves. Another study applied a self-supervised DINO to over 10,000 PAS-stained images from 384 biopsy slides [16]. For disease classification – including minimal change disease (MCD), mesangial proliferative glomerulonephritis (MPGN), membranous nephropathy (MN), and DN – this method achieved an AUROC of 0.93 and an area under the precision-recall curve (AUPRC) of 0.82, outperforming a standard supervised ImageNet-pretrained fine-tuned model (AUROC 0.89, AUPRC 0.76). External validation on two independent datasets confirmed DINO’s superiority, particularly when labeled data were limited.

Electron microscopy (EM) remains indispensable for visualizing ultrastructural features that are not apparent by light microscopy. A multicenter study developed an automated system called TEM-AID (transmission EM image-based AI-assistance device) using YOLO-v8 detection, the segment anything model, and a human-in-the-loop mechanism [17]. It achieved excellent segmentation performance, with a mean intersection-over-union of 0.835 and a mean Dice coefficient of 0.874 for detection of glomerular basement membranes, foot processes, and electron-dense deposits. For subtype classification of seven glomerular diseases, including MCD, MN, DN, mesangial proliferative glomerulonephritis, IgA nephropathy (IgAN), lupus nephritis (LN), and thin basement membrane nephropathy, the human-AI comparison test involving 454 patients and four pathologists found that TEM-AID alone had an accuracy of 0.886 and AUROC of 0.963, exceeding the mean unaided pathologist accuracy of 0.724. Importantly, the most clinically relevant finding was that human-AI collaboration performed the best with TEM-AID assistance on diagnosing or classifying the seven diseases; pathologist accuracy improved by approximately 12% on average. Key limitations include the need for prospective validation in non-Chinese populations and better interpretability.

#### Prognostic Applications

Deep learning has also been used to derive prognostic information from biopsy images. In one study on IgAN, a ResNet-18 CNN was used to develop a deep learning-based predictive score [18]. For predicting 5-year kidney failure (defined as the need for dialysis or kidney transplant), this score performed as well as the gold standard Oxford classification of the International IgA Nephropathy Prediction Tool (IIgAN-PT) and the clinical decision support system, achieving an AUROC of 0.84 vs. 0.77 and 0.74, respectively; accuracy of 0.82 vs. 0.77 and 0.71, and an F1 of 0.67 vs. 0.61 and 0.56. The model also identified prognostically relevant features not captured by standard scoring, such as inflammation within areas of interstitial fibrosis and the number of certain casts. Prospective, multicenter validation, and integration with clinical data remains necessary.

### Data-Driven Phenotyping

Unsupervised ML methods help discover latent patterns in complex data, which are valuable for identifying distinct disease subtypes within heterogeneous glomerular diseases [9].

#### Membranous Nephropathy

Unsupervised clustering was used to classify 178 PLA2R-positive MN patients into three clinical phenotypes based on 25 clinicopathological variables [19]. One cluster had mild disease (low anti-PLA2R, creatinine, proteinuria, and triglycerides) and achieved the best outcomes (high remission rate and low risk of ESKD or death). Another cluster was characterized by high body mass index, proteinuria, and inflammation markers; despite intensive immunosuppressive therapy, it had the worst outcomes (ESKD or death). High anti-PLA2R (>100 U/mL) and proteinuria (>8 g/24 h) predicted poor remission. These results suggest moving from a standard treatment approach to personalized, phenotype-guided management in MN.

#### IgA Nephropathy

A multicenter study of 2,000 IgAN patients used principal component analysis and k-means clustering on 28 clinical and pathological variables to identify four subgroups [20]. Patients with advanced CKD and severe chronic pathologic damage benefited from renin-angiotensin system blocker therapy (HR 0.16 for ESKD; *p* < 0.0001) but not steroids. By contrast, patients with nephrotic-range proteinuria and active inflammatory lesions responded well to steroid monotherapy (HR 0.19 for ESKD, *p* = 0.005). However, the retrospective design, Chinese-only population, and non-randomized treatment assignments limit generalizability and increase the risk of bias.

#### Thrombotic Microangiopathy

Thrombotic Microangiopathy (TMA) is a heterogeneous disorder that often involves the kidneys. Although renal histology can help identify TMA, it does not reliably distinguish underlying causes. Moubarak et al. [21] integratively clustered 64 clinical and histologic variables from 283 biopsy-proven TMA cases and revealed three distinct phenotypes suggestive of immune, toxic, or severe vascular-lesion drivers. This data-driven classification helps prioritize diagnostic and therapeutic considerations, providing a foundation for prospective validation and molecular profiling. These studies show that unsupervised ML can reveal meaningful patient subgroups with different outcomes and treatment responses, supporting a precision-medicine approach.

### Advancing Diagnosis and Differentiation

ML has been applied to improve diagnostic accuracy across several glomerular diseases using clinical, serologic, imaging, and molecular data.

#### Focal Segmental Glomerulosclerosis

Several approaches aim to distinguish focal segmental glomerulosclerosis (FSGS) without biopsy. One study profiled 5,416 plasma proteins in 232 Korean patients with FSGS, IgAN, MCD, or MN [22]. Using elastic net regression, the model achieved AUROCs >0.8 for distinguishing MCD, MN, and IgAN but correctly identified only 21% of FSGS cases. Limitations include the exclusion of advanced CKD (eGFR <45 mL/min/1.73 m^2^), an East Asian-only cohort, and FSGS’s inherent heterogeneity.

#### Membranous Nephropathy

A deep learning model DenseNet161 with logistic regression fusion combining light microscopy and immunofluorescence achieved excellent diagnostic performance for MN, with an F1 of 0.973, better than using either imaging type alone (F1 approximately 0.93 for each) [23]. Another immunofluorescence-based model distinguished MN, IgAN, DN, and controls with a macro-F1 of 0.971, performing particularly well for MN (precision 0.989, recall 0.997, F1 0.993), outperforming individual pathologists (F1 range 0.916–0.966). Model-pathologist agreement was high (Cohen’s k = 0.974). In a diagnostic test of 138 patients with various glomerular diseases, the system correctly identified 30 of 43 MN cases (69.8% diagnostic rate) [24]. These findings highlight the complementary value of integrating multiple imaging modalities.

Beyond imaging, a random forest classifier using glomerular gene expression distinguished MN from MCD, FSGS, and IgAN with an AUROC of 0.91 [25]. Tubulointerstitial expression performed poorly (AUROC 0.50), indicating a glomerular-specific signature. Top predictive genes were validated in independent European cohorts. This study identified 158 MN-specific genes (120 validated) involved in ion transport, cell structure, and wound healing. This molecular characterization enables patient stratification and reveals potential therapeutic targets.

#### IgA Nephropathy

Several noninvasive diagnostic models have been developed for IgAN. One study compared 5 ML models plus logistic regression in 1,268 patients [26]. Light gradient boosting machine (LightGBM) achieved the highest AUROC (0.913) in the derivation cohort, while eXtreme gradient boosting (XGBoost) performed well in temporal validation (AUROC 0.894). SHAP (SHapley Additive ExPlanations) analysis identified age, serum albumin, IgA/C3 ratio, and urine red blood cells as key predictors. All models showed good calibration with Brier scores 0.107–0.131. This study highlighted both the strength of tree-based methods for tabular clinical data and the value of interpretable post hoc explanation tools such as SHAP.

Another study reported that a random forest model using clinical variables from 602 patients achieved 0.823 accuracy and an AUROC of 0.89 on an independent test set, substantially outperforming physician judgment (AUROC 0.59) [27]. Notably, elevated IgG was identified as a discriminating factor, challenging the traditional focus on IgA alone.

#### Lupus Nephritis

Noninvasive ML models have also performed well in LN. For diagnosing LN in systemic lupus erythematosus patients, one XGBoost model using features including antistreptolysin, retinol binding protein, lupus anticoagulant, and proteinuria achieved an AUROC of 0.995, an AUPRC of 1.000, and a balanced accuracy of 0.990, outperforming LightGBM (AUROC 0.992) and random forest (AUROC 0.989) [28]. Another study using nine ML models to predict proliferative LN found that random forest performed best (AUROC 0.880), with key predictors including traditional markers (anti-dsDNA, complement, creatinine) and less conventional ones (serum chloride, neutrophil percentage, cystatin C, urinary pH, and IgM) [29].

Together, these studies show that ML can enhance diagnostic accuracy by integrating diverse data types, potentially reducing delays and invasive procedures. ML also holds promise for uncovering novel biomarkers and pathways, deepening our understanding of disease biology and informing future therapies.

#### Prediction Modeling and Risk Stratification

ML has also been applied to refine risk prediction models across glomerular diseases, representing a meaningful advance in prognostic assessment [30]. The most informative studies are those that move beyond discrimination alone to evaluate calibration, validation strategy, and potential clinical actionability.

#### MCD and FSGS

A LASSO (the least absolute shrinkage and selection operator) regression model using four routine variables (mean arterial pressure, serum albumin, uric acid, and lymphocyte counts) predicted acute kidney injury in MCD, achieving AUROCs of 0.84 in training and 0.75 in validation, with DCA confirming clinical net benefit [31].

The NEPTUNE study of 242 MCD/FSGS patients evaluated 12 EM features and identified six distinct EM clusters with different outcomes [32]. The “classic MCD” cluster (effacement and microvillus transformation) had the highest proteinuria remission rates, while the “endothelial cell changes” cluster (podocyte and endothelial damage) had the highest progression rates (≥40% eGFR decline or ESKD) and lowest remission. Limitations include modest interrater reproducibility for some descriptors (requiring averaged scores from multiple pathologists, impractical clinically) and small sample size limiting statistical power for subgroup analyses and extensive adjustment.

Separately, another NEPTUNE study quantified 37 glomerular features and identified three morphologic clusters with different outcomes [33]. The worst-outcome cluster had more FSGS (89%) and African American patients (48%), lowest baseline eGFR (54 mL/min/1.73 m^2^) and showed steepest eGFR decline (−5.5 mL/min/1.73 m^2^/year) with lowest remission and highest progression rates. Notably, 1,920 differentially expressed genes distinguished this cluster, with enrichment of immune/inflammation pathways. Clusters did not fully align with conventional diagnoses, highlighting that current classification misses clinically relevant heterogeneity.

Zee et al. [34] used the same cohort to develop random forest models using histologic and ultrastructural features, achieving AUROCs of 0.9 for progression, 0.85 for remission, and 0.81 for treatment response. Adding computationally derived tubular features to clinical data improved the AUROC for disease progression from 0.753 to 0.811 and for remission from 0.738 to 0.772; however, external validation showed more modest gains [35].

The proposed Focal segmental glomerulosclerosis Nephro Decision Support System (FNDSS) integrates multiple ML algorithms to cover the entire FSGS management pathway, rather than focusing on a single prediction task [36]. However, it was trained on synthetic rather than real-world data, which may not capture clinical heterogeneity, and lacks external validation in prospective, multicenter cohorts.

#### Membranous Nephropathy

In MN, a LightGBM model using 59 variables predicted the composite outcome (ESKD or doubling of serum creatinine) with an AUROC of 0.892, accuracy of 0.909, recall of 0.741, and F1 of 0.905 [37]. SHAP analysis revealed that high anti-PLA2R and low IgG4 deposition were the strongest risk predictors. Another study used LASSO regression and support vector machines (SVMs)-recursive feature elimination to identify seven ferroptosis-related genes (*NR1D1*, *YTHDC2*, *EGR1*, *ZFP36*, *RRM2*, *RELA*, *PDK4*) with diagnostic value for MN (AUROCs 0.681–0.855), offering insights into pathogenesis and potential therapeutic targets [38].

#### IgA Nephropathy

An early random forest model using six basic predictors (sex, age, hypertension, creatinine, proteinuria, and histological grading) achieved an AUROC of 0.926 for predicting ESKD in IgAN; adding Oxford MEST pathology scores raised the AUROC to 0.961, adding C3 staining raised it to 0.973, and adding selected laboratory variables (eGFR, blood urea nitrogen, uric acid, hemoglobin, albumin) increased it to 0.955–0.966 [39]. This study was the first to apply an interpretable random forest model to predict ESKD in a large Asian IgAN cohort, addressing the black-box limitations of ANNs. However, limitations included lack of direct model comparison, multi-time-point assessment, time-varying treatment adjustment, short follow-up, and selection bias (e.g., excluded patients with eGFR <15 mL/min/1.73 m^2^).

A subsequent XGBoost model achieved a C-statistic of 0.89 (derivation) and 0.84 (validation) for predicting ESKD or 50% eGFR decline; a simplified three-variable score (proteinuria, global glomerulosclerosis, and interstitial fibrosis and tubular atrophy [IFTA]) had good discrimination (C-statistic 0.80–0.81) and excellent calibration (Hosmer-Lemeshow *p* = 0.8) [40]. Limitations include Chinese-only data, exclusion of eGFR <30 mL/min/1.73 m^2^ or proteinuria <0.5 g/24 h, variable treatments, and a combined endpoint.

An ANN-based tool predicted 5- and 10-year ESKD with AUROCs of 0.82 and 0.89, high sensitivity (0.92 and 0.89), accuracy (0.80 and 0.83), and discriminant Harrell C-indices (0.81 and 0.86). In an external testing cohort, the tool correctly predicted ESKD in 91% of IgAN patients [41]. A recent study in high-risk IgAN patients (CKD stage 3–4 with proteinuria ≥1 g/24 h) compared several survival models and found random forest performed best for predicting ESKD or death, with C-indices of 0.871 (training) and 0.810 (test). LASSO and random forest identified six key predictors: blood urea nitrogen, uric acid, serum potassium, glomerulosclerosis ratio, hemoglobin, and IFTA [42].

Recently, Hölscher et al. [43] evaluated whether deep learning-based pathomics features (quantitative morphometric biomarkers from digitized PAS-stained biopsies) could replace or improve upon traditional MEST-C scoring. The results suggest that the main advantage of pathomics is not incremental predictive power but rather its potential as a high-throughput, objective, and reproducible alternative to traditional histopathology scoring. Notably, the predominantly white European cohort limits generalizability to diverse populations with known ethnic differences in IgAN morphology and progression.

#### Lupus Nephritis

An early ANN model moderately predicted LN development in patients with systemic lupus erythematosus, achieving 0.687 accuracy, 0.738 sensitivity, and 0.63 specificity, with the positive predictive value of 69.2% and negative predictive value of 68%, still outperforming logistic regression and clinicians [44]. For predicting renal flare, an XGBoost model using 59 variables achieved C-indices of 0.822 in the derivation cohort and 0.819 in the validation cohort. A simplified six-variable score (partial remission, endocapillary hypercellularity, age <28 years at remission, serum albumin <35 g/L at remission, positive anti-dsDNA at remission, and low serum C3) achieved C-indices of 0.747 in the training and 0.746 in the validation, with acceptable calibration (Hosmer-Lemeshow *p* = 0.35 and 0.62) [45].

In class IV±V LN patients, a random survival forest model predicted adverse outcomes (≥50% eGFR decline, ESKD, or death) with C-indices of 0.950 (training) and 0.771 (testing), outperforming XGBoost and Cox regression. It also showed better time-dependent AUROCs (e.g., 0.965 at 12 months, 0.984 at 36 months, 0.972 at 60 months), lower Brier score (0.144), improved net reclassification improvement and integrated discrimination improvement, and greater clinical net benefit on DCA [46].

#### Diabetic Kidney Disease

In patients with type 2 diabetes, a comparison of 7 ML methods found that SVM and random forest performed best for predicting DKD development (eGFR <60 mL/min/1.73 m^2^) [47]. This study identified ten clinical factors (e.g., age, blood pressure, lipids) and five genetic markers linked to inflammation and lipids. It was the first to systematically compare seven different ML methods for predicting DKD using both clinical and genetic data, while excluding obvious kidney markers (urinary albumin-to-creatinine ratio, serum creatinine) and treatment data to uncover novel predictive patterns. Another study using a convolutional autoencoder on EHR data from over 64,000 patients predicted 6-month DKD aggravation (progression from stage 1 to stage 2 or higher based on urinary protein) with an average AUROC of 0.743 and accuracy of 0.71; performance improved when longitudinal data were added [48].

Together, these studies show that ML enables more accurate risk stratification across glomerular diseases by integrating phenotypic, genotypic, and temporal data. By capturing complex patterns in clinical, pathological, biomarker, and longitudinal EHR datasets, ML can identify at-risk patients early, supporting timely interventions to prevent or delay long-term renal complications. Incorporating ML-based tools into clinical guidelines is a key step toward precision nephrology.

### Treatment Response Prediction

Predicting how individual patients will respond to treatment is a highly valuable but challenging use of ML in glomerular disease. By identifying who is most likely to benefit from specific therapies, ML models can help guide treatment choices, reduce unnecessary immunosuppression, and advance personalized medicine.

#### Membranous Nephropathy

In refractory MN, a logistic regression model using five factors (higher albumin, lower creatinine, lower proteinuria, higher eGFR, and lower anti-PLA2R) predicted rituximab response (complete or partial remission) with an AUROC of 0.99 (sensitivity 0.938, specificity 0.979) [49]. This study addresses a critical need as refractory MN patients have few options and high risk of kidney failure, but limitations include single-center, Chinese-only, and retrospective design, which may limit generalizability and introduce bias. In a prospective study, a multivariate regression model incorporating systemic inflammation response index and B cell levels improved prediction of rituximab response compared to traditional markers (proteinuria, albumin, anti-PLA2R) alone, achieving an AUROC of 0.86 vs. 0.81 [50].

A deep learning model (LightGBM) incorporating pathological signatures from whole-slide images achieved AUROCs of 0.85 (training) and 0.77 (validation) for predicting treatment response in patients with MN receiving standardized immunotherapy (low-dose prednisolone combined with cyclophosphamide for 6 months), whereas a clinical-only model performed worse (AUROCs 0.62–0.75) [51]. However, 95% of patients were anti-PLA2R positive in this study, limiting generalizability to PLA2R-negative patients. PLA2R antibody levels and baseline proteinuria – both key predictors in MN – were excluded from the model.

Another study developed a dynamic online nomogram to predict 2-year non-remission of proteinuria in idiopathic MN using four baseline clinical predictors: disease duration ≥6 months (indicating greater chronicity), 24-h urinary protein, D-dimer, and serum anti-PLA2R [52]. Random forest achieved the best performance (AUROC 0.869), with sensitivity 0.700 and specificity 0.897, outperforming XGBoost (0.734) and logistic regression (0.710). The nomogram demonstrated good discrimination (C-index: 0.835 training, 0.874 validation), calibration (mean absolute error 0.047), and clinical utility (DCA 10–88%). An online tool provides individualized risk estimates. Nevertheless, the retrospective design, Chinese-only population, modest sample size, and absence of pathologic and tubular injury variables remain important limitations.

#### IgA Nephropathy

One study showed that personalized risk assessment using the IIgAN-PT improved treatment decisions compared to using proteinuria alone [53]. DCA showed a net benefit difference of 23.4% at a 40% risk threshold, and the tool reduced unnecessary treatment by up to 35.1%. Using proteinuria ≥1 g/24 h (current KDIGO guideline) had negative net benefit above an 18% risk threshold, indicating overtreatment of low-risk patients. But its limitations should be noted: the tool predicts risk only at biopsy (not over longer follow-up), may not generalize to non-Asian/Caucasian groups, assumes immediate treatment (guidelines recommend initial conservative care), and real-world impact remains unassessed.

A subsequent study found that higher glomerular macrophage infiltration strongly predicted complete or partial remission to immunosuppression (13–40-fold increased probability) in high-risk IgAN patients (persistent proteinuria ≥1 g/24 h after 3 months of renin-angiotensin system blocker); a six-variable model including these cells achieved AUROCs of 0.87 in both training and validation cohorts, with good calibration (Hosmer-Lemeshow *p* = 0.13) [54]. This prospective study provides a practical tool to identify responders and reduce unnecessary treatment, but limitations of single-center, specialized immunohistochemistry requirement and Chinese-only population (mycophenolate mofetil efficacy not proven in non-Chinese patients) may restrict its generalizability.

A personalized decision support system using an auto-encoder model achieved an AUROC of 0.91 (sensitivity 0.46, specificity 0.96) for predicting IgAN prognosis (ESKD, kidney transplant, death, or 50% eGFR decline), outperforming network biomarkers with graph feature engineering (AUROC 0.90) and random forest with molecular biomarkers (AUROC 0.83) [55]. By treatment, AUROCs reached 0.95 (supportive care), 0.96 (glucocorticoids), and 1.0 (immunosuppressants). The systems aligned with nephrologists in 97 of 100 validation cases. The prospective design strengthens reliability, but the 4-year follow-up for endpoint may be insufficient for slow-progressing IgAN. Other limitations include the following: low sensitivity (0.46) means missing >50% progressors; perfect AUROC (1.00) raises overfitting concerns (small sample size, no external validation); complex method requires specialized resources; and Chinese-only population limits generalizability.

#### Lupus Nephritis

A random forest model combining traditional markers (C3, C4, creatinine, anti-dsDNA, proteinuria) with urine biomarkers (chemokines, cytokines) achieved an AUROC of 0.79 (sensitivity 0.76, specificity 0.73) for predicting 1-year complete response to induction therapy in LN, substantially outperforming clinical markers alone (AUROC 0.61) [56]. But two limitations need to be highlighted: there was no external validation (though internal testing showed no overfitting) and the outcome required a normal eGFR (90 mL/min/1.73 m^2^), which is irrelevant for patients with baseline impairment.

Another study combined biopsy features with routine laboratories across 5 ML models (logistic regression, random forest, SVM-linear, SVM-Gaussian, ANN), achieving cross-validated AUROCs >0.75 (average sensitivity 0.70, specificity 0.73, positive predictive value 0.71, and negative predictive value 0.71) for predicting poor 1-year treatment response in LN [57]. Interstitial inflammation was the most consistent predictor across all models, highlighting the importance of tubulointerstitial pathology – often overlooked compared to glomerular features.

A third study used clinical data plus 13 urine biomarkers to predict 1-year LN treatment response through SVM-linear, random forest, and logistic regression models. Adding urine biomarkers improved AUROCs for eGFR response from 0.67–0.76 to 0.73–0.84; external validation AUROCs reached 0.80–0.92 [58]. Consistent key variables across algorithms (race, baseline eGFR, baseline urine protein-creatinine ratio, CXCL8, pentraxin) and external validation (though small) strengthen the findings. Nevertheless, not all flares were biopsy confirmed, treatments differed across subcohorts, long-term outcomes beyond 1 year were unavailable, and no prospective trial has tested whether use of the model improves care.

A LASSO model using hub genes predicted treatment response after renal flare (AUROC 0.949; in validation, accuracy 0.86, precision 0.83) and identified lymphocyte-specific protein tyrosine kinase as a key regulatory gene linked to T-cell infiltrations [59]. Incorporating biological phenotypes into ML improves both transparency and predictive performance. However, the use of different transcriptomic platforms reduces generalizability. Use of only 1 ML algorithm and no experimental verification of lymphocyte-specific protein tyrosine kinase weakens its reliability.

A deep learning model using four types of whole-slide image stains predicted 12-month complete response (proteinuria <0.5 g/24 h with normal or near-normal eGFR) to induction therapy in LN, with internal AUROC of 0.901 and external AUROC of 0.840 – outperforming conventional clinical and pathology parameters (eGFR, chronicity index, and 3-month proteinuria reduction) [60]. Key histologic features included tertiary lymphoid structures, glomerulosclerosis, and IFTA. This is the first study to apply deep learning across all four routine stains and make AI predictions interpretable via visualization. Limitations include retrospective design over 2 decades (evolving care standards), only cyclophosphamide or mycophenolate mofetil as induction therapy (applicability to other regimens unknown).

## Clinical Deployment Successes, Failures, and Lessons Learned

Although previous sections highlight promising ML applications, real-world clinical deployment remains rare. Successful models share key features: free accessibility, simplicity, and authoritative endorsement. The IIgAN-PT – externally validated and incorporated into KDIGO guidelines – exemplifies this success [61]. Other Web-based tools for risk stratification and treatment response prediction exist [20, 40, 45, 52, 57], but most are limited by retrospective, single-center Chinese data, lack of external validation, and unproven clinical impact. Thus, these tools require further validation before widespread adoption.

Failures have revealed important lessons. A large review found that over 80% of validation studies reported lower discrimination than the original derivation dataset, with AUROC dropping by a median of 11% – largely due to overfitting, especially as models became more complex [62]. Most omit calibration, making risk estimates unreliable. Deployment failures also stem from domain shift, lack of workflow integration, and opaque user interfaces [63]. To date, few, if any, ML models in glomerular disease have demonstrated improvements in patient outcomes in randomized clinical trials. Key lessons include discrimination alone is insufficient; calibration and DCA are essential; multicenter external validation is necessary; simpler models are more readily adopted; prospective testing of human-AI interaction is required; and current models should serve as decision support tools with continuous monitoring for bias.

## Challenges and Ethical Considerations

Despite technical progress, several challenges must be addressed before ML can be widely adopted [64]. Feasible solutions for each challenge are discussed and summarized in online supplementary Table 6.

### Data Scarcity, Biases, and Generalizability

Large, high-quality datasets remain scarce, especially for rare glomerular conditions, limiting generalizability and increasing the risk of overfitting. Data heterogeneity across institutions causes domain shift, where models perform poorly outside their training environment. Missing data, irregular follow-up, and outcome misclassification add further difficulty. The PROBAST + AI tool provides a standardized framework for assessing bias risk across four domains: study population, predictors, outcomes, and statistical methods [65]. A systematic review using PROBAST + AI to evaluate AI models for predicting pediatric immunization non-adherence found that no included study conducted external validation, highlighting a common gap [66]. For missing data, methods such as multiple imputation by chained equations and K-nearest neighbors imputation should be reported according to TRIPOD + AI guidelines [6].

### Interpretability

Complex models often function as “black boxes,” making it difficult for clinicians to understand predictions or assess alignment with clinical reasoning. Calibration errors can lead to misestimation of absolute risk, affecting critical decisions. The TRIPOD + AI guidelines provide checklists for ML-specific reporting, including model interpretability [6]. Interpretability can also be enhanced using SHAP analysis, calibration plots, and concept bottleneck models that first predict human-understandable features.

### Security and Privacy

Multimodal datasets often contain potentially identifiable elements that cannot be fully anonymized without losing some utility. Combining data across modalities can increase reidentification risk compared to single-modality datasets. A framework is recommended for multimodal selective de-identification that uses prompt-driven targeting to anonymize personally identifiable information while preserving clinical utility [67]. Accordingly, secure storage, encrypted transfer, rigorous access controls, and auditable data-use policies are essential. Federated learning enables multicenter training without data sharing, and synthetic data generation offers promising solutions but requires technical sophistication and oversight [68]. One study from COVID-19-associated AKI showed that federated models outperformed local models, with the greatest gains at sites with smaller datasets [69].

### Ethical Issues

Automated treatment recommendations raise complex ethical concerns [64]. Clinicians may place undue trust in algorithmic outputs, and calibration errors or unrecognized bias could result in inappropriate therapy choices. A systematic review of empirical studies on AI ethics in healthcare identified six categories of ethical issues related to transparency and trust, bias and fairness, privacy and data security, accountability and responsibility, ethical and moral concerns, and regulatory and legal issues [70]. Ethical deployment requires adherence to ethical frameworks, bias mitigation, post-deployment monitoring and auditing, and stakeholder involvement in governance [70]. While no solution is perfect, the most feasible immediate steps are policy based: journals should mandate external validation and adherence to reporting guidelines, and institutions should require clinician confirmation before acting on any AI-generated recommendation.

## Future Directions

### Multimodal Data Fusion

Integrating digital pathology, multi-omics, clinical data, and imaging is a major frontier for precision nephrology [23, 71]. Future models will combine whole-slide images, EM, molecular profiles, and laboratory data into comprehensive risk assessments. This approach may uncover disease endotypes – biologically distinct subgroups within traditional diagnostic categories – that respond differently to specific therapies. Both horizontal integration (combining multiple data types from the same patient) and vertical integration (discovering new patterns across data modalities) will shape next generation of ML tools in glomerular disease [24].

### Clinical Workflow Integration

For ML to impact patient care, its outputs must fit seamlessly into clinical workflows. Embedding validated models into EHR systems can enable real-time risk estimation, automated alerts, and visual displays of disease trajectories. Well-designed interfaces can support treatment discussions and shared decision-making [72].

Rigorous validation should precede clinical deployment. However, demanding external validation for all models without considering feasibility is impractical. Awaiting perfect validation may delay potential useful tools, whereas deploying poorly validated may expose patients to harm. A more realistic approach is a risk-stratified validation framework in which low-stakes assistive tools may be introduced with close monitoring, while high-stakes treatment recommendations require stronger external and prospective evidence (online suppl. Material 2). Once models are implemented, ongoing monitoring is essential to evaluate real-world clinical impact, including care quality, efficiency, and patient outcomes, with periodic retraining as needed.

### Large Language Models

Large language models (LLMs), such as ChatGPT, represent a specialized deep learning application focused on generating novel content and are being widely explored in nephrology [73]. One recent study evaluating GPT-4o and GPT-5 for feature extraction from pathology reports found error rates of about 5% for simple cases and 10% for complex cases, with high expert agreement (κ > 0.9) [74]. Another multimodal model combining LLM-extracted information with numerical preoperative data predicted acute kidney injury after cardiopulmonary bypass with an AUROC of 0.92, outperforming random forest (AUROC 0.59) and logistic regression (AUROC 0.60); external validation yielded an AUROC of 0.91 [75]. Similarly, Bobart et al. [76] developed a PubMed BERT-based natural language processing model to extract primary diagnoses from free-text native kidney biopsy reports in a diverse cohort of 2,666 patients with 3,042 reports. The model achieved excellent overall performance, with an average AUROC of 0.95 across all 42 diagnosis types, improving to 0.97 for the 20 most common diagnoses. These findings highlight the growing potential of LLMs to enhance diagnostic accuracy and risk prediction in kidney disease.

## Conclusion

ML is becoming increasingly relevant to biomedical research and clinical practice, and its relevance to glomerular disease is growing rapidly. By enabling deeper phenotyping, more accurate risk stratification, and personalized therapeutic approaches, ML provides a powerful framework to address the inherent complexity and heterogeneity of these disorders. However, it should be highlighted that ML is designed to augment, not replace, the nephrologist’s expertise.

Nevertheless, realizing this potential will require intentional action. We therefore conclude with a call to action and five specific priorities for the field. First, models must undergo rigorous external validation and calibration assessment, with journals enforcing the TRIPOD + AI guidelines. Second, shared, diverse, multicenter datasets should be built, particularly for rare diseases. Third, interpretability and clinically actionable risk thresholds need to be integrated from the start of model development. Fourth, prospective implementation studies, including randomized trials, must be conducted. Fifth, continuous monitoring for performance drift and bias should be embedded after deployment. For clinicians, the immediate action is to critically appraise ML studies using TRIPOD + AI. For researchers, the priority is multicenter collaboration and prospective validation. For journals and funders, the responsibility is to enforce reporting standards and prioritize clinically actionable models. The path forward requires collective will and rigorous execution.

## Acknowledgment

The authors acknowledge the large and growing body of excellent research on machine learning in glomerular diseases. Space limitations preclude citation of many such studies, and the authors recognize that many important contributions are not referenced in this review.

## Conflict of Interest Statement

The authors have no conflicts of interest to declare.

## Funding Sources

This study was not supported by any sponsor or funder.

## Author Contributions

M.J.V.B. drafted and revised the manuscript. R.P.S., W.C., and C.T. revised the manuscript. J.M. conceptualized, revised, and finalized the manuscript. All authors approved the final version of the manuscript.
